# 

**Published:** 1902-08-30

**Authors:** 


### NOTICE TO CORRESPONDENTS.

All MSS., Letters, Books for Review, and other matters intended for the Editor should be addressed The Editor, "The Hospital," The Hospital Building, 28 & 29 Southampton Street, Strand, W.C.

The Editor cannot undertake to return rejected MSS., even when accompanied by stamped directed envelope.

### Notice to Advertisers and Subscribers.

All Advertisements, Orders for Copies of The Hospital, and Business Communications should be addressed to **THE MANAGER, (Not to the Editor)**, The Hospital, 28 & 29 Southampton Street, Strand, London, W.C.

The Cost of Subscription, post free, is as follows.—Per week, 2½d.; per quarter, 2s. 8d.; per half-year, 5s. 3d.; per annum, 10s. 6d. Foreign:—Per annum, 15s. 2d., payable, in all cases, in advance.

**NOTICE.—Vol. XXXI. of "The Hospital" (October 1901, to March 1902), in handsome cloth binding is now on sale at the office of this Journal, price, 7s. 6d. Cases for binding the Numbers contained in this Volume 1s. 6d. Index to Vol. XXXI., 2½d., post free.**

**The Monthly Part for September will be ready on September 1. Price 6d., by post 8d.**

**The Students' Number of "The Hospital" will be issued next week, September 6.**

### BOOKS RECEIVED.

Baillière, Tindall and Cox.

"Hydatid Disease of the Lungs." By Alfred Austin Lendon, M.D. (Lond.)

His Majesty's Stationery Office.

"Supplement to the Thirtieth Annual Report of the Local Government Board, containing the Report of the Medical Officer for 1900-01."

Ch. Eggiman and Cie, Genève.

"Typhlite, Perityphlite, Apdendicite." Par le Dr. Bourget.

J. B. Lippincott Co.

"International Clinics." Vol. I. 1902.

John Wright and Co.

"Medical Ethics: A Guide to Professional Conduct." By Robert Saundby, M.D.Edin.

### The Student of Medicine.

### APPOINTMENTS.

**E. Bertram Appleby**, M.B., B.S.Durh., appointed Senior Resident Surgeon to the Nottingham General Dispensary. **F. W. J. Coaker**, L.R.C.P.Lond., M.R.C.S., appointed Medical Officer of Health for the Bromsgrove Rural District. **R. J. Collie**, M.D.Aberd., appointed Medical Examiner to the London County Council, and Assistant Medical Officer to the London School Board. **David Ewart**, M.B., Ch.B.Edin., F.R.C.S.E., appointed Honorary Surgeon to West Sussex, East Hampshire, and Chichester General Infirmary. **W. R. Fenton**, B.A., M.B. Univ. Dublin, appointed Acting Principal Medical Officer in Mashonaland. **G. W. H. French**, F.R.C.S., appointed Assistant Surgeon to St. Paul's Hospital for Skin and Genito-Urinary Diseases, Red Lion Square, W.C. **W. J. Hill**, L.R.C.P.Lond., M.R.C.S., appointed Medical Officer of Health to the Clevedon Urban District Council. **G. H. Jenkins**, L.R.C.P., L.R.C.S.Edin., L.F.P.S.Glasg., reappointed Medical Officer of Health to the Usk Urban District Council, Mon. **Fred. Wm. Dobson McGachen**, M.D., D.P.H.Lond., appointed Medical Officer to the Weymouth and Westham District, and Public Vaccinator Weymouth and Melcombe Regis. **W. E. Peacock**, M.D.Durh., appointed Medical Officer of Health to the Felling Urban District Council. **J. W. Thomson Walker**, M.B.Edin., F.R.C.S., appointed Assistant Surgeon to the North-West London Hospital. **John Wharton**, B.A., B.C.Cantab., appointed House Surgeon to the Manchester Eye Hospital.

**"The Hospital" Institutional Library.**

"Hospitals and Asylums of the World." 4 Vols., with a Portfolio of Plans, complete £12 12s.

"Burdett's Hospitals and Charities, 1902." 5s.

"Cottage Hospitals, General, Fever, and Convalescent." 10s. 6d.

"Hospital Expenditure: The Commissariat." 2s. 6d.

"A Legal Handbook for the Use of Hospital Authorities." 2s. 6d.

"The Cottage Hospital Case Book or Register of Patients." 10s. and 12s. 6d.

"The Furnishing and Appliances of a Cottage Hospital." 6d.

All these are published by the SCIENTIFIC PRESS, Ltd., and may be obtained through any bookseller, or direct from the publishers, 28 and 29 Southampton Street, Strand, London, W.C.

